# Design and Evaluation of a Wireless Sensor Network Based Aircraft Strength Testing System

**DOI:** 10.3390/s90604195

**Published:** 2009-06-03

**Authors:** Jian Wu, Shenfang Yuan, Genyuan Zhou, Sai Ji, Zilong Wang, Yang Wang

**Affiliations:** 1 The Aeronautic Key Laboratory for Smart Materials and Structures, Nanjing University of Aeronautics and Astronautics, 29# Yu Dao Street, Nanjing, China, 210016; E-Mails: wujian@nuaa.edu.cn (J.W.); hjfd641xsc@yahoo.com.cn (Z.W.); wy52119514@163.com (Y.W.); 2 Jinangsu Teachers University of Technology, 1801# Zhongwu Road, Changzhou, China, 213001; E-Mail: zgy@jstu.edu.cn (G.Z.); 3 Nanjing University of Information Science and Technology, 219# Ningliu Road, Nanjing, China, 210044; E-Mail: 000507@nuist.edu.cn (S.J.)

**Keywords:** aircraft strength testing, wireless sensor network, cluster-star topology protocol, undercarriage static test

## Abstract

The verification of aerospace structures, including full-scale fatigue and static test programs, is essential for structure strength design and evaluation. However, the current overall ground strength testing systems employ a large number of wires for communication among sensors and data acquisition facilities. The centralized data processing makes test programs lack efficiency and intelligence. Wireless sensor network (WSN) technology might be expected to address the limitations of cable-based aeronautical ground testing systems. This paper presents a wireless sensor network based aircraft strength testing (AST) system design and its evaluation on a real aircraft specimen. In this paper, a miniature, high-precision, and shock-proof wireless sensor node is designed for multi-channel strain gauge signal conditioning and monitoring. A cluster-star network topology protocol and application layer interface are designed in detail. To verify the functionality of the designed wireless sensor network for strength testing capability, a multi-point WSN based AST system is developed for static testing of a real aircraft undercarriage. Based on the designed wireless sensor nodes, the wireless sensor network is deployed to gather, process, and transmit strain gauge signals and monitor results under different static test loads. This paper shows the efficiency of the wireless sensor network based AST system, compared to a conventional AST system.

## 1. Introduction

Although the accuracy of the existing numerical codes in aerospace structure simulation is increasing steadily, Aircraft Strength Testing (AST) is still considered the preferred means for reliable simulation. Airframe and component strength testing is used to measure and analyze structure parameters and performance (e.g., stress, displacement, vibration amplitude, and fatigue life) for the evaluation and validation of structure mechanical properties and theory design. Fatigue and static tests in ground testing facilities are one of the most important means of research of aircraft structure strength. Traditionally, the cable-based AST systems for aircraft structures usually involve large numbers of wires employed for communication among sensors and centralized data acquisition systems. These wires on specimen structures can be cumbersome, which brings high installation costs and inefficient maintenance. As sensors have no means to locally process their data, the centralized data server is responsible for the aggregation, storage and processing of all measurement data. If AST systems include hundreds, or even thousands, of testing sensors, the testing task computations in a centralized testing system can become burdensome and time-consuming.

In response to the cost and performance shortcomings of centralized cable-based AST systems, this paper present an exploration of wireless sensor network (WSN) technology for adoption in AST systems. In recent years, WSN has been applied in many engineering fields, ranging from national defense and military affairs, to behavior observation of animals, structural health monitoring, traffic controls, medical treatment and sanitation and disaster monitoring. Volgyesi *et al.* [1] proposed a decentralized method of shooter localization and weapon classification using soldier-wearable networked sensors. Straser and Kiremidjian [2] were the first to describe algorithms for structural health monitoring using wireless sensors. Lynch *et al.* [3] designed a low-cost wireless sensing unit for deployment as the wireless structural monitoring system on the Alamosa Canyon Bridge. Xu *et al.* [4] described the design and evaluation of a wireless sensor network system called Wisden for structural data acquisition based on the Mica-2 motes. Yuan *et al.* [5] and Zhao *et al.* [6] developed a composite structural health monitoring system base on the Mica wireless platform and multi-agent technology. However, there has been no similar research related to WSNs designed for AST applications.

The research in this paper aims to develop a WSN based aircraft AST system design. A small-size, high-precision, and shock-proof wireless sensor node is designed for multi-channel strain gauge signal conditioning and monitoring. To address the need for low-power consumption, timeliness, and scalable operations, a cluster-star network topology protocol is adopted and researched. The application layer interface is designed in detail. To verify the functionality of the designed wireless sensor network for distribution AST capability, a multi-point distribution testing system is developed for static tests of a real aircraft undercarriage. The experimental results prove the advantages of the wireless sensor network based AST system, compared to the conventional cable-based AST system.

## 2. AST System Analysis and WSN Framework

**Figure 1.**
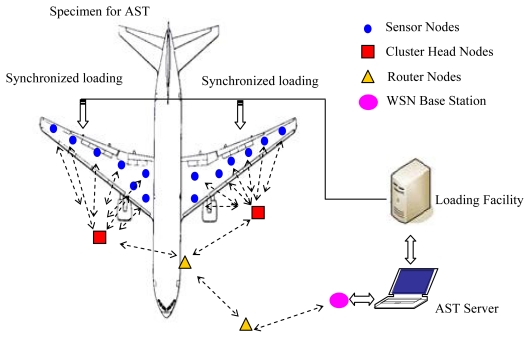
The WSN framework for AST.

As shown in Figure 1, this paper presents a WSN framework for AST systems. The WSN based AST systems consists of a number of sensor nodes, several cluster head nodes, and additional optional wireless router nodes that help with data aggregation and transmission via wireless multi-hop. Each cluster head node associates some sensor node to create its own subnet for AST implementation in a certain area of the specimen. The monitoring and control of the WSN measurement system must be simultaneous with the AST loading facility. The next sections prove that this WSN framework could completely support low-power, multi-point, and heterogeneous operations with a distributed synchronization mechanism.

In order to design an efficient WSN system based AST, it is important to understand the critical parameters and design requirements such as testing realizability, timeliness, scalability, and energy efficiency.

- Structure strain changes under different testing loads are the main testing parameter in the fatigue and static tests. Because these testing results are used to evaluate the mechanical properties of the aircraft structure, the WSN based AST system should have sufficient precision for strain measurement, e.g. ±0.1%. For large-scale specimens or a full-scale testing, the number of testing sensors could reach several hundred, therefore, every sensor node should be designed with multi-sensor input channels. In addition, WSN hardware systems must have anti-EMI capability, for the electromagnetic interference (EMI) resulting from other field equipment and the environment could adversely affect overall WSN measurements.
- Real-time data acquisition and transmission of strains at different sites on a specimen when a load is applied during the test are essential for realization of the testing function. When the testing engineer might want to query real-time data from some specific nodes to estimate the current status of the particular testing area of a specimen, features might be added to allow breaches in normal network operation to transmit control signals back to the sensing nodes. This could help in eliminating manual node debugging operations and extending testing functions.
- Over the duration of testing, some sensing nodes may fail or their batteries may become depleted. Also, a need may arise for installation of more sensing nodes to monitor particular processes and equipment more closely and precisely. The WSN should be scalable to accommodate changes in the number of nodes without affecting the entire system operation.
- Sensor nodes are autonomous devices that usually derive their power from a battery mounted on each node. It becomes necessary to have an inherent energy-saving means in every component of the WSN system to prolong the lifetime of each node in the network. All layers of the architecture are thus required to have built-in power awareness. DC power might also be used in the AST system to provide the energy for the WSN measurements, so a flexible energy supply should be designed for the WSN system based AST.

## 3. Design and Implementation of the WSN Based AST System

### 3.1. High-Precision Wireless Strain Node Design

**Figure 2.**
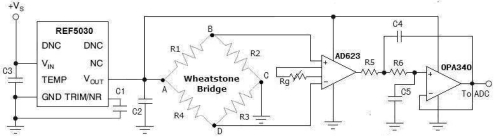
The schematic circuit diagram for high-precision strain measurements.

The fundamental objective of the wireless sensor network based AST system is the design of a dedicated high-precision wireless strain sensor node. High-precision means the testing random error is small and replicated measurements can provide closely similar results. In this paper, this design aims to achieve a node testing accuracy of ±0.1%. The wireless sensor node in this paper is designed with integrated bridge voltage circuit which enables precise strain measurement, so the resistance strain gauges might be directly connected to the designed wireless sensor nodes and need no other additional instruments. In order to have good testing precision, the bridge voltage provided to the Wheatstone-bridge must have enough accuracy. Because the strain measurement has low-voltage and varying-load features, the series reference scheme is selected for better initial tolerance, temperature coefficient and power dissipation than using shunt reference [7]. Specially, a series reference IC, REF5030, is used for providing the constant voltage for the Wheatstone-bridge circuit. The REF5030 is able to provide a 3V high precision power with excellent temperature drift (3 ppm/°C) and high accuracy (0.05%). The bridge circuit output corresponds to structural strain monitored. Since the sensitivity of strain gauge is low, the instrumentation amplifier AD623 is adopted to amplifier the bridge circuit output. As a low-power instrumentation amplifier, the AD623 can offer excellent accuracy. The maximal input offset drift of AD623 is no more than 2 μV/°C. The maximal supply current of AD623 is no more than 575 μA. Since strain gauges are usually adopted to monitor static signals, a low-pass filter is designed to eliminate the high frequency noise. The voltage follower is adopted to output the filtered voltage signal. Figure 2 shows the schematic circuit diagram for the designed high-precision strain measurement.

A 10-bit analog-to-digital (A/D) converter with a sampling rate of 15 KSPS integrated in an Atmel Mega128 MCU is adopted directly in the node design. It has eight multiplexed single ended input channels. Detailed design information on the processing core is outlined in our previous paper [8]. Specifically, in the wireless communication design a TI CC2420 RF transceiver is chosen instead of a CC1000 for the communication capability improvement provided. The CC2420 is a true single-chip 2.4 GHz IEEE 802.15.4 compliant RF transceiver designed for low-power and low-voltage wireless applications. With 2.4 GHz RF frequency and -25 dBm output power, the wireless transceiver only draws 8.5 mA of current while actively transmitting, guaranteeing the low-power characteristics of the designed wireless node. The circuits of the WSN node are divided to be manufactured on three four-layer printed circuit boards. A dedicated installation box is designed to attach these boards. The reason for adopting the four-layer circuits and divided design is to sufficiently separate the analog and digital circuit components. The other benefit obtained from the design is that each part can easily be upgraded according to different application requirements.

**Figure 3.**
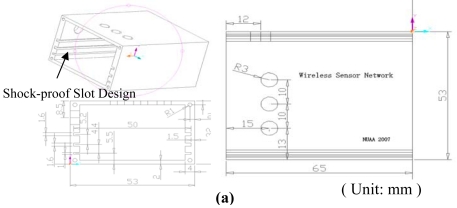

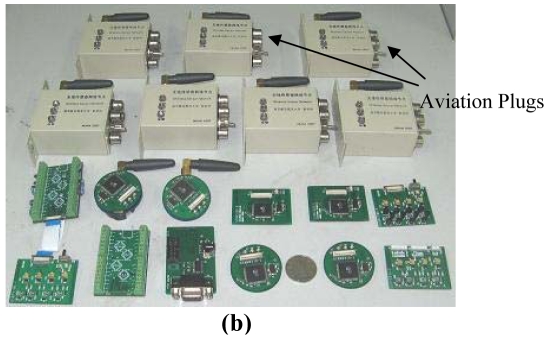
(a) Encapsulation size chart. (b) Designed node and shock-proof encapsulation.

In order to make the WSN hardware systems light-weight, easy-assemble and anti-EMI, this paper designed an all-aluminum shock-proof encapsulation for the WSN measurement in AST applications. Figure 3(a) shows the aluminum encapsulation size chart with dedicated shock-proof slot design. Figure 3(b) shows the picture of the designed wireless sensor node and the encapsulation for lightweight installation. All the interfaces of the encapsulation use reliable aviation plugs. The power to supply the wireless sensor node is designed to use 5 V direct current (DC) power since all the components are low-power and the 5 V DC power is easy to use in AST systems. Thus, four normal AA batteries can power the complete wireless sensor node.

### 3.2. Wireless Sensor Network Node Performance Tests

**Figure 4.**
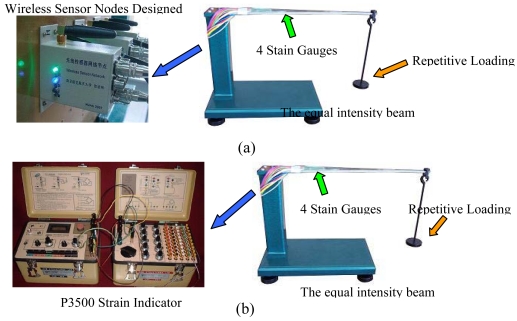
The picture of tests setup for static error and repeatability error. (a) Tests for output voltage under repetitive Loadings. (b) Tests for strain changes under repetitive Loadings.

**Figure 5.**
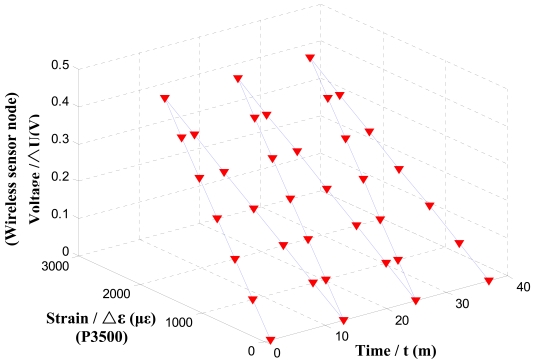
The wireless sensor node output voltage VS. P3500 strain readings with 1 minute increments.

After the fabrication of the sensor node, node performance tests were conducted. The first test was to evaluate the static error and repeatability error of the designed node. Four stain gauges are attached to the upper and lower exterior of the equal intensity beam. The strain gauges are connected to the wireless sensor node. A P3500 strain indicator is used to calibrate the WSN sensor node. The P3500 strain indicator is a portable instrument for stress analysis testing with strain gauge based transducers. Figure 4 shows the calibration setup. Table 1 shows the experimental results of three loading cycles with positive and negative loading. The plots of the sampled voltage values of the wireless senor node with respect to three cyclic load strains measured by the P3500 are shown in Figure 5. These plots clearly show the tested strain changes for every repetitive loading cycle.

The static error for the wireless sensor node can be represented by γ given by:

(1) $$\gamma =\pm \frac{3}{y_{FS}}\sqrt{\frac{1}{n-1}\sum_{i=1}^{n}{\left(\Delta y_{i}\right)}^{2}}\times 100\%$$

where, Δ*y_i_* is the residual difference for the test points, n is the number of the test points, and *y_FS_* is the full scale output. Using the test data in Table 1 to the formula (1), a static error γ ≈ ±0.1% can be achieved for the wireless sensor node. Experiments show the developed WSN node is accurate and meet the pre-design requirements.

The other two tests include the wireless sensor node service lifetime and transmission range. Table 2 shows the different service lifetime tests to corresponding AA battery capacity. The service lifetime is divided by active time and low-power time. The transmission period is set to 10 seconds. Power supply load for the active mode is about 40 mA in this test configuration. It can be calculated by totalizing the operation currents of three functional modules. The node total current consumption in the low-power mode is about 100 μA with the sensor input unit shut-down mode, so a conservative estimation for the anticipated life of the wireless sensor node using four batteries (each 2,000 mAh) is over two years. Table 3 shows the different transmission ranges corresponding to different transmission powers.

**Figure 6.**
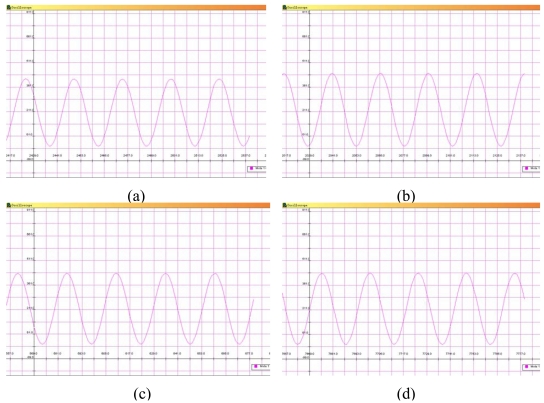
The received signals of the wireless sensor node in the test environment set by (a) no EMI factors, (b) a Bluetooth phone, (c) a GSM mobile phone and (d) five body obstacle.

The final performance test of the wireless sensor node was its anti-EMI capabilities. The function generator is used to simulate the sensor input signal, and is connected directly to the ADC input channel of the wireless sensor node. A sine wave with 250 mV the peak value is generated for the test input signal. The test wireless sensor node collects the wave signal and transmits a data packet per second with the radio channel set to 2,400 MHz. Some EMI factors such as Bluetooth devices, mobile phones and human bodies were considered in the anti-EMI test. Figure 6 shows the signals received by another immobile node connected to the monitor server by the serial 232 port when the EMI environment is set by no EMI factors adding, a Bluetooth phone call, a GSM mobile phone call and human body obstacle separately. From the received signal, it can be found that data can be collected and transmitted correctly in the EMI environment.

### 3.3. The Distributed Synchronization Protocol for AST Measurement

Since the designed wireless sensor nodes support IEEE 802.15.4/ZigBee [9,10] implementation, this paper presents a reliable cluster-star network topology protocol for distribution AST applications to ensure real-time data acquisition and transmission when different loads are applied. The IEEE 802.15.4/Zigbee protocol stack is being considered as a promising technology for low-cost low-power wireless sensor networks. However, the current IEEE 802.15.4/Zigbee specifications restrict the synchronization in the beacon-enabled mode to star-based networks which only support simple small wireless synchronization applications [11].

**Figure 7.**
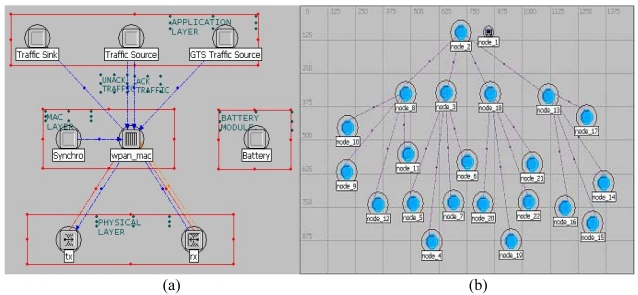
(a) The cluster-star network node model. (b) Simulation experiments setup.

**Figure 8.**
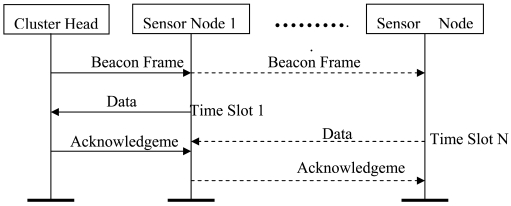
Data transmission sequence chart.

**Figure 9.**
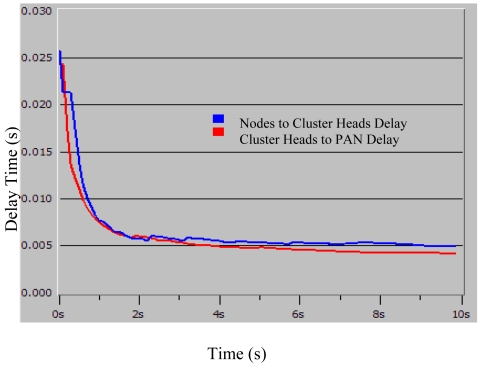
The simulation result for network delay with 16 nodes and four cluster heads.

The cluster-star network topology adopted for scalable wireless sensor network based AST system is shown in Figure 1. The main advantage of the star topology is its simplicity for synchronization. The cluster-star topology has only a PAN coordinator; however, it differs from the star topology in that all nodes make up a two layer framework. In the clusters, cluster heads can communicate with any other device within its radio range to a star topology. In the top layer, the unique PAN coordinator only communicates with the cluster heads to a star topology. The topology can completely support low-power, multi-point, and heterogeneous operations with a distributed synchronization mechanism. With the Guaranteed Time Slot (GTS) mechanism and beacon-enabled mode, the cluster-star network topology protocol is modeled on the OPNET Modeler [12]. Figure 7 (a) shows the cluster-star network node model. In order to validate the function of the protocol, the simulation is conducted by 16 nodes and four cluster heads. Figure 7 (b) shows the simulation experiments setup. The adopted distributed synchronization mechanism is based on beacons and acknowledgments frames. The active time of the clustered WSN is divided to some time slots which are dedicated to package transmission in only one link every time. The CSMA/CA scheme is used to reliably send command and data frames. Because the sent frame requires an acknowledgment, the sender must wait for it before sending a new message. The wait or the retransmission mechanism consists on a timer that is activated after a transmission that requires an acknowledgment. The synchronized data transmission sequence chart is shown in Figure 8. Figure 9 shows the simulation result for network delay with 16 nodes and four cluster heads. The average times of end-to-end delay in the two layer framework both approximate 5 milliseconds.

### 3.4. WSN Application Layer Design for AST

The WSN application layer design for AST aims to synchronously control and monitor AST programs. During the strength testing experiments, WSN measurements should be controlled to ensure simultaneous data acquisition of all the strain gauges when a load is applied to the specimen. When the testing engineer wants to query real-time data from some specific nodes to estimate the current status of the particular testing area of a specimen, the WSN application layer interface should allow breaches in normal network operation to transmit control signals back to the sensing nodes. In addition, because some sensing nodes may fail or more sensing nodes may be added to the system over the duration of testing, the WSN application layer interface should be scalable to accommodate changes in number of nodes without affecting the entire system operation. Finally, although the coordinator node and the cluster heads could be supplied by the fixed power, strain sensor nodes are autonomous devices that usually derive their power from a battery mounted on each node. WSN application layer interface should be designed to prolong the lifetime of WSN by effective active and sleep controlling mechanism.

**Figure 10.**
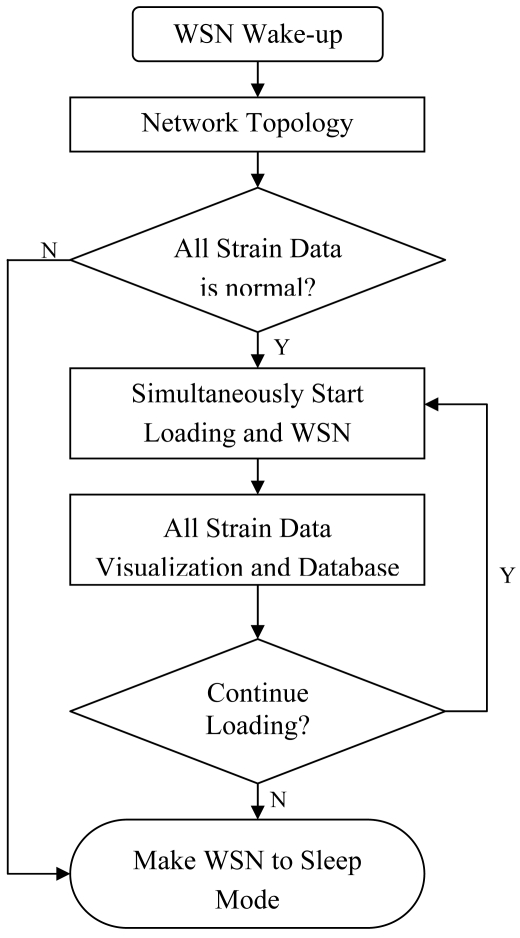
The flow chart for the WSN application layer design.

**Figure 11.**
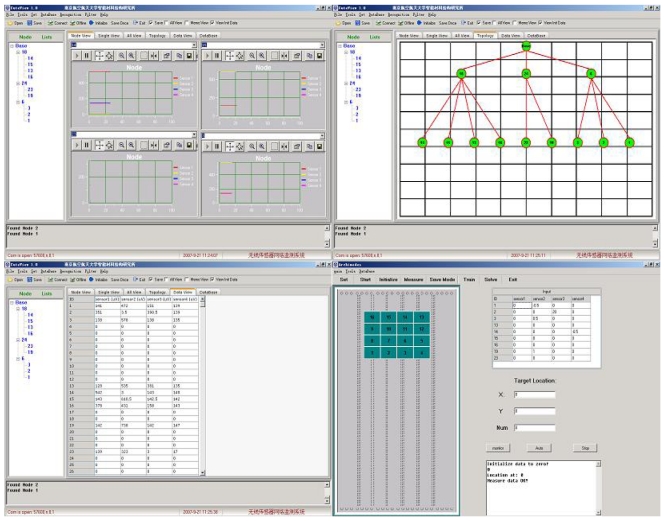
The WSN application layer interface for AST.

In view of the above considerations, a WSN application layer interface was designed for AST measurement programs. The WSN measurement process for AST includes the following steps: when the user tasks are allocated in the AST application layer interface, the WSN will be waked up for the specific measurement. The user tasks might be triggered periodically or by testing engineers who want to query real-time data from some specific nodes for the particular testing areas of a specimen. Then the base station node send the wake-up instruction to the particular cluster heads which will awaken the own cluster members. After the initialization of the WSN, the relevant network topology will be visualized in the interface. Based on the real-time data transmission scheme, all collected strain data will be gathered to the base station. If no abnormal sensor data or failure nodes exist, the sensor nodes will collect the sensor values with the testing load synchronously. Finally the AST application layer interface will aggregate all testing data and show the integrated result about the specific specimen. The flow chart for the WSN measurement is shown in Figure 10. Figure 11 shows the overview of the WSN application layer interface for AST.

## 4. Evaluation Research on a Real Aircraft Structure

**Figure 12.**
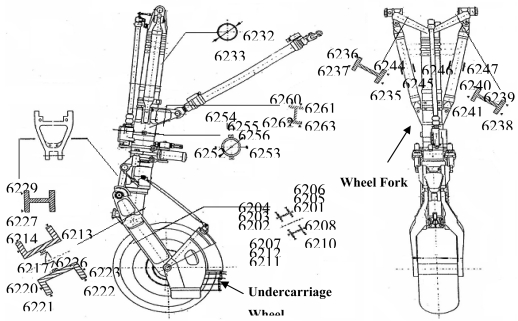
The real aircraft fore-undercarriage of a fighter plane and the strain gauges distribution.

**Figure 13.**
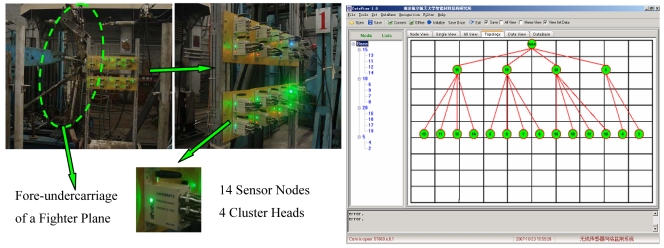
(a) Field setup of the wireless sensor network system. (b) Monitoring interface of the network system.

In order to validate the capability of the designed WSN based AST systems, in this paper a multi-point system is developed for a real aircraft undercarriage for static testing in Xian Aircraft Strength & Research Institute of China. Figure 12 shows the real aircraft fore-undercarriage of a fighter plane and the strain gauge distribution. In the three axes of the fore-undercarriage wheel and two axes of the wheel fork, 43 deployed strain gauges were chosen for the static test and connected to the wireless sensor nodes. Figure 13 (a) shows the field setup of the wireless sensor network system which involves 14 sensor nodes and four cluster heads. Table 4 shows the correspondence of connection between 43 strain gauges and 14 wireless sensor nodes. Figure 13 (b) shows the monitoring interface of the network system which is controlled simultaneously with the static test loading facility.

**Figure 14.**
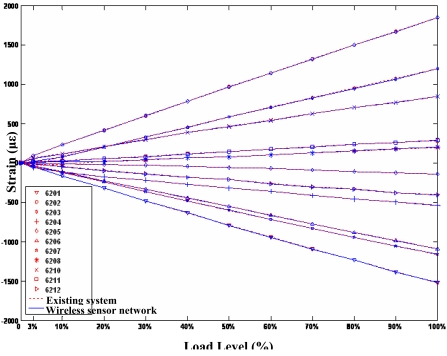
Test data comparison between WSN and the cable-based system.

**Figure 15.**
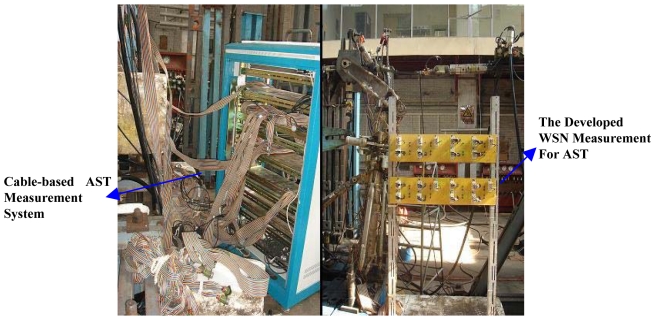
Setup comparison between WSN and the cable-based system for AST.

Before the setup of the WSN based AST system, the existing cable-based measurement system was been used to static test the real aircraft fore-undercarriage, and the testing data was saved for drawing a capability comparison between the existing measurement system and the WSN system. Figure 14 shows the test data comparison between the WSN based AST system and the existing cable-based measurement system. At the same testing point, the maximal difference between the cable-based measurement data and the WSN testing data is no more than 10 με. In figure 14, the two testing curves for the same testing point almost coincide. Therefore, the test results in Figure 14 show that the developed WSN system can be successfully used for structural strength test. Figure 15 shows their system setup comparison. The evaluation experiment shows the WSN based AST technology can obviously reduce the number of wires employed. The overall cost of the WSN based AST system in this paper is nearly one-tenth of the existing wired system in Xian Aircraft Strength & Research Institute of China. Because of the processing capability of wireless sensor nodes, the efficiency of the WSN system deployment and debugging in this evaluation experiment is evidently higher than the wired system. For example, the deployment time for the WSN system in this paper is about two hours, whereas the wired system needs almost five hour to be deployed.

## 5. Conclusions

Though the WSN based AST system presented in this paper is proven to be successful for static testing, the system design will be much more complicated when the number of testing points to be measured increases greatly. Furthermore, fatigue testing for full-scale structure requires higher data transmission rates, data synchronization and data buffer processing capacity. Therefore, hardware capabilities for the WSN based AST systems should be improved in the further research. Networking and routing protocols should be also studied deeply to solve these problems.

## Figures and Tables

**Table 1.** Experimental data of the sensor node by repetitive loading.

**Load Conditions / ΔG (N)**	**Output Voltage (V) / Strain (με)**					

	**Cycle 1**		**Cycle 2**		**Cycle 3**	

	**Plus Direction**	**Negative Direction**	**Plus Direction**	**Negative Direction**	**Plus Direction**	**Negative Direction**

**0**	1.144/0	1.144/0	1.144/0	1.143/0	1.143/0	1.143/0
**2**	1.218/387	1.218/387	1.218/387	1.218/387	1.218/387	1.218/386
**4**	1.293/769	1.293/769	1.293/770	1.293/769	1.292/769	1.292/769
**6**	1.367/1151	1.367/1151	1.367/1150	1.367/1151	1.368/1150	1.367/1151
**8**	1.442/1532	1.442/1532	1.442/1532	1.442/1532	1.442/1532	1.443/1532
**10**	1.517/1918	1.516/1913	1.517/1915	1.516/1915	1.517/1915	1.517/1915
**12**	1.591/2290	1.591/2290	1.590/2291	1.590/2291	1.591/2294	1.591/2294

**Table 2.** Different lifetime to corresponding AA batteries.

**Each Battery (mA·h)**	**Lifetime (h)**
1,000	185
2,000	364
3,000	588

**Table 3.** The different transmission ranges corresponds to the transmission power.

	**Test1**	**Test2**	**Test3**	**Test4**
Power	-20	-10	0	10
Range (m)	10.1	16.5	29	75

**Table 4.** The correspondence of connection between 43 strain gauges and 14 wireless sensor nodes.

**Nodes ID**	**2**	**4**	**6**	**7**	**8**	**9**	**11**	**12**	**13**	**14**	**16**	**17**	**18**	**19**
Strain Gauges ID	6201 6202 6203 6204	6205 6206 6207 6208	6210 6211 6212 6213	6214 6217 6220	6221 6222 6223	6263	6226 6227	6229 6232	6233 6235 6236	6237 6238 6239	6240 6241 6244 6245	6246 6247 6252	6253 6254 6255 6256	6260 6261 6262
